# Scaling laws in geo-located Twitter data

**DOI:** 10.1371/journal.pone.0218454

**Published:** 2019-07-24

**Authors:** Rudy Arthur, Hywel T. P. Williams

**Affiliations:** Social & Environmental Data Analysis Lab, Department of Computer Science, University of Exeter, Exeter, United Kingdom; Purdue University, UNITED STATES

## Abstract

Twitter has become an important platform for geo-spatial analyses, providing high-volume spatial data on a wide variety of social processes. Understanding the relationship between population density and Twitter activity is therefore of key importance. This study reports a systematic relationship between population density and Twitter use. Number of tweets, number of users and population per unit area are related by power law functions with exponents greater than one. These relations are consistent with each other and hold across a range of spatial scales. This implies that population density can accurately predict Twitter activity, but importantly, it also implies that correct predictions are not given by a naive linear scaling analysis. The observed super-linearity has implications for any spatial analyses performed with Twitter data and is important for understanding the relationship between Twitter use and demographics. For example, the robustness of this relationship means that we can identify ‘anomalous’ geographic areas that deviate from the observed trend, identifying several towns with high/low usage relative to expectation; using the scaling relationship we are able to show that these anomalies are not caused by age structure, as has been previously proposed. Proper consideration of this scaling relationship will improve robustness in future geo-spatial studies using Twitter.

## Introduction

Twitter is an important source of data for a variety of academic fields, including many that explore relationships between social behaviours (manifested on Twitter) and spatial/geographic features (identified by geo-location of Twitter users and content). Yet there remains a lack of understanding around the key underpinning relationship between Twitter usage and population density, which is a primary determinant of many social phenomena.

In this work we show there is a power law relationship between population density and social media activity which is consistent across a range of spatial scales. This has important implications for any large-scale spatial analyses of social media data, e.g. normalising background activity for event detection, or accounting for common co-variates in demographic studies. We show that controlling for this non-linear relationship destroys the apparent correlation, for example, between proportion of people under 35 and social media activity. Finally, in the course of our analysis we find that GPS-tagged tweets originate from a highly atypical set of sources when compared to other tweets. Given the frequency with which geo-tagged tweets are used as a basis for spatial analysis in the literature, this is a significant finding by itself.

**Fig 1:**
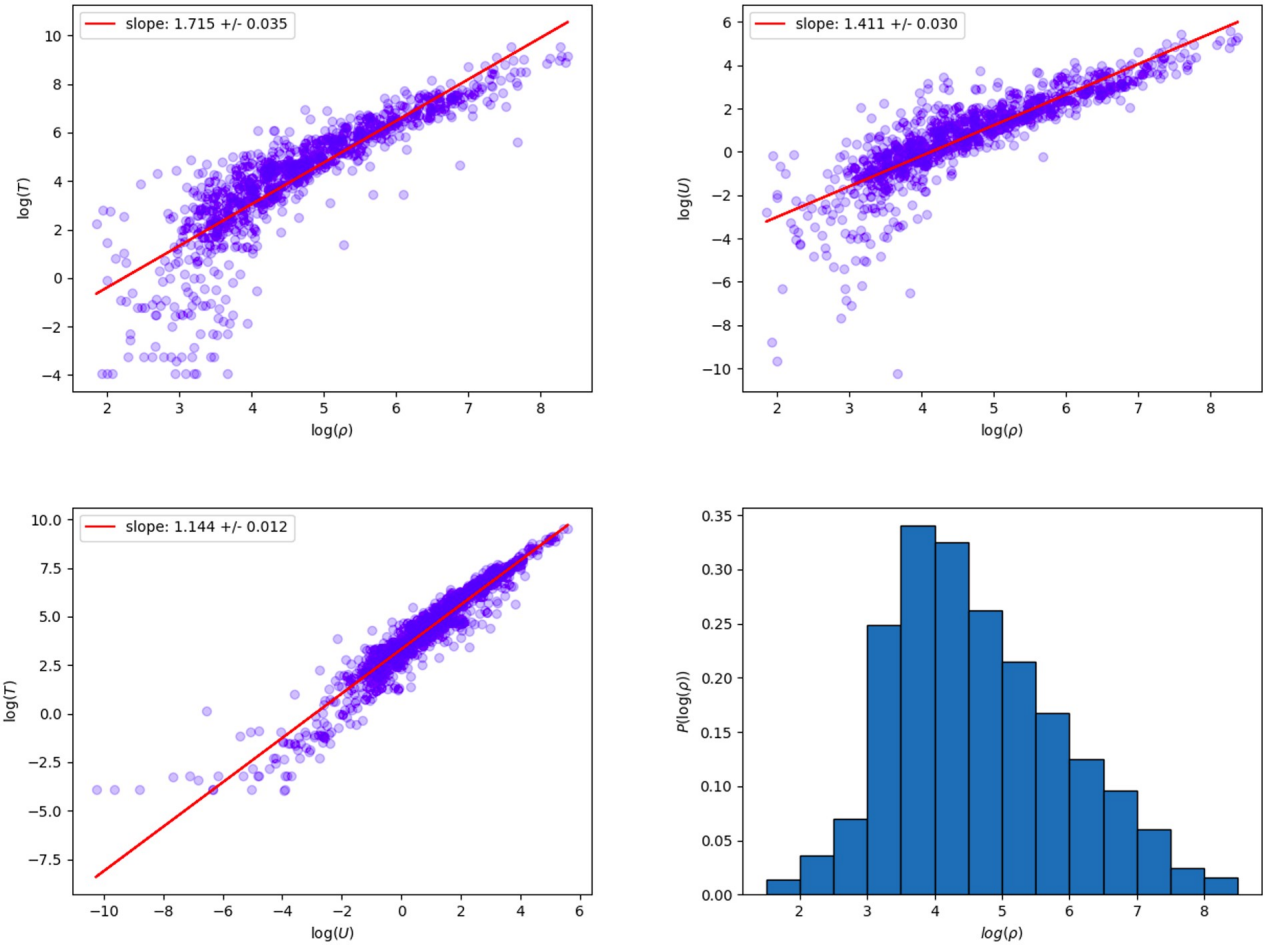
Scaling laws for place-tagged tweets. Left: Tweet density *T* versus population density *ρ*, slope of the line is *α*. Middle: User density *U* versus population density *ρ*, slope of the line is *β*. Right: Tweet density *T* versus user density *U*, slope of the line is *γ*. Using a 40 × 40 grid.

**Fig 2:**
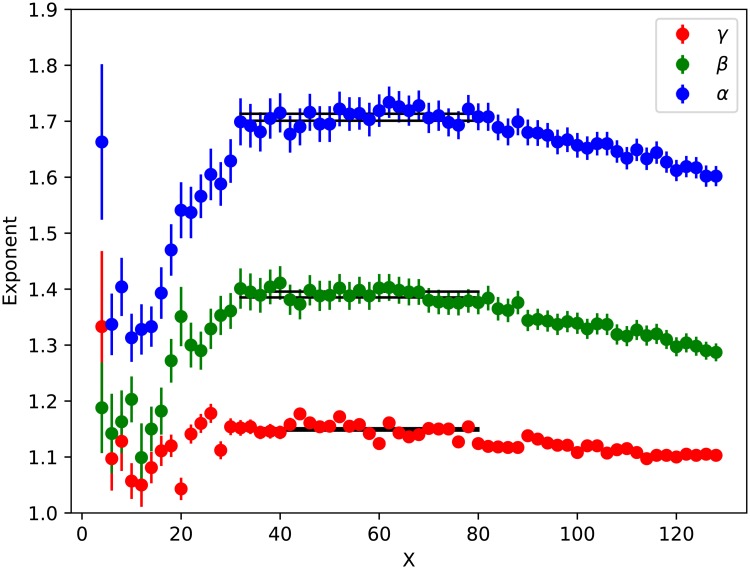
Exponents *α*, *β*, *γ* versus grid size *X* for place-tagged tweets. The horizontal lines mark the largest range of grid sizes across which the exponents agree within errors, showing the average value of the exponent across the range.

The outline of this paper is as follows. We begin with a short survey of related work in **Background** and then outline our approach to collecting and aggregating tweets in Section **Materials and methods**. Our main results related to scaling laws are shown in Figs 1 and 2 which show the scaling relationships in place-tagged tweets. The section **Checking the consistency relation** verifies the consistency of the scaling relationships across a range of measurement scales and their self consistency according to Eq 4. The section **Finding Anomalies** explores when and why these scaling relationships fail to hold. Our second main result is in this section, which explores the local anomalies that remain in observed Twitter activity after accounting for the effects of population density. We then show that the density of young people, argued elsewhere to be a factor in the amount of Twitter activity, has no additional effect once the common correlation of both variables with population density is removed. We conclude in the **Discussion** section where we explore the significance of our findings and the potential for future work.

## Background

Twitter is a social media platform whose open access API makes it very popular with researchers. Twitter data has been used extensively in the disaster response literature to study earthquakes [1], wildfires [2], floods [3] as well as real time disaster related communication [4], [5]. Twitter has also been used to study social phenomena like human mobility [6] language [7], land use patterns [8], public health [9], happiness [10] and many other topics. With around 328 million users as of August 2017, [11] Twitter is now established as a key data source in quantitative social science and for geographic applications such as event detection and regional comparisons, with some suggesting that it is perhaps even overused [12]. Given this, it is important to understand demographic biases like age, gender, ethnicity and to understand the relationship between population density and volume of Twitter activity, in order to normalise event detection algorithms. A lot of work has been done on this topic already, see [13] [14] [15] [16].

Studies of demographic bias find users in cities and urban areas over-represented in collections of Twitter data. Mislove et. al. [13] found “…Twitter users are more likely to live within populous counties than would be expected from the Census data, and that sparsely populated regions of the US are significantly underrepresented.” Hecht et. al. [14] find that urban users are over-represented in geo-located social media, and also provide more information than rural users. Longley et. al. [15] find Twitter in London is not representative of the true age profile or gender ratio but representation of ethnicity is more reflective of the true population. They also find a fairly strong level of ethnic segregation among Twitter users in London. More recent work by Malik et. al. [16] on US Twitter usage has identified other demographic variables such as higher median income, being in an urban area or having more young people as being predictive of more geolocated tweets originating from an area. To our knowledge, no one has studied the systematic relationship between population density and Twitter activity. Mislove et. al. [13] plot Twitter representation rate against population for US counties. However they do not explore this relationship in depth, nor determine its functional form. Neither do they take the crucial step of accounting for population *density*, by taking county area into consideration, as we do in this work.

It is the aim of this paper to determine how Twitter usage is related to population density. In particular, we are motivated by work of Takhteyev et. al. [17] and Stephens et. al. [18] which shows that a user’s connections are not randomly distributed around the globe, but rather that Twitter builds on existing social structures (e.g. neighbourhoods, cities, airline connections, languages) and does not supersede them. The work of Tizzoni et. al. [19] is very interesting in this regard. In building a model of human interactions as a reaction-diffusion process on a graph, they construct a graph of Twitter users, using mentions to connect users in the same metropolitan area. They find that the connectivity (rescaled cumulative degree, the total number of links times the proportion of the population who are Twitter users) scales super-linearly with population size.

The search for systematic effects with population density and urban bias of Twitter usage relates closely to work on scaling laws in cities [20] [21] [22] [23] and patterns of human mobility [24, 25]. Scaling laws are power law relationships that describe the variation in some quantity with population, population density or some other size metric. For example the amount of food consumed is a linear function of the population, while the amount of electrical cable is a sub-linear function of population density (since denser neighbourhoods require fewer connections to service the same number of people). Many creative or social outputs like wages or number of patents scale super-linearly with city size. The work of [26], similarly to [19], showed that social networks (this time constructed from mobile phone data) also scale super-linearly with city size.

Social media activity, here Twitter accounts and tweets, is the output of a social network. Thus, like patents, wages or other social outputs, according to the scaling law literature we would expect their number to grow super-linearly with population. The work of [27] and [28] who show similar effects using cities as the units of analysis and total population as a size metric. Unlike many studies of scaling laws e.g. [20] we use population density instead of total population. Our sampling area contains not only cities, but medium sized towns, villages and very rural or wilderness areas. The work of Arcaute et. al. [29], [30] shows that the precise definition of *city* is crucial and simple changes can cause apparent scaling relationships to vanish. By using population density we avoid the issue of defining a city and can use arbitrary sampling areas. This allows us to vary the spatial sampling distribution, so that our results do not depend on particular definitions of *city* or *town*, as well as to vary the size of the sampling areas, showing us the range of spatial scales where our results hold. This makes our results more robust, given they don’t depend on the specifics of the sampling procedure, and reliable, given we can see where they apply and where they break down.

The literature on the science of cities also provides important guidance on how to deal with non-linearity. The work of Bettencourt [21] and others [31] shows that deviations from the non-linear trend are the correct quantities to use when it comes to ranking and correlating variables which scale super-linearly with size. We will show that this is also true when considering Twitter activity and simple demographic variables.

## Materials and methods

### Tweet collection

We collected tweets from the South-West UK by passing a geographical bounding box with longitude from -5.8 to -1.2 and latitude from 49.9 to 52.2 to the Twitter Streaming API. All data was gathered in compliance with all applicable Twitter API policies and terms of use [32] and in accordance with Twitter’s Developer Policy [33]. As with any work of this nature, the choice of sampling area is somewhat arbitrary, so long as the area chosen is reasonably representative. The South-West UK was a sample of convenience, since the authors maintain an ongoing collection of tweets from the area. The region is representative of the UK, with a population of around 9.6 million people, and contains a mixture of rural and industrial areas, as well as large cities (Bristol, Southampton, Cardiff). Thus the South-West UK is not atypical or anomalous, making it a reasonable sample for this study.

The collection ran from 11/4/2016 to 10/4/2018 with some gaps in the collection (due to machine downtime) covering all of December 2016 and all of March 2017. The Twitter Streaming API is free of cost and easy to access compared to the full Twitter datastream. This makes it the usual choice for research purposes. The Streaming API is rate limited, only allowing us to collect a restricted number of tweets, at most 1% of all the tweets on Twitter [34]. Since we are looking at a small area relative to the (theoretically) global coverage of the Twitter platform, and only collecting tweets with geo-location tags, the threshold for rate limiting will only rarely, if ever, be crossed in this study and the Streaming API should be sufficient for a general survey of tweets.

We use two metadata properties of each tweet to locate it: geo-tags and place-tags. Geo-tags are GPS co-ordinates added by the user’s mobile device that give a precise location for a particular tweet. According to the Twitter API documentation [35]: “Places are specific, named locations with corresponding geo coordinates. They can be attached to tweets by specifying a place_id when tweeting. Tweets associated with places are not necessarily issued from that location but could also potentially be about that location.” Place-tags are often quite precise and all of our tweets have a populated “place” field. Place-tags are added to the tweets of a user who opts in to using Twitter’s location services. Once a user opts in to location services, e.g. by tagging a tweet in a certain place, all subsequent tweets will automatically include a general location label [36] as a place-tag.

We will show geo-tagged tweets are a minority (as other studies have found e.g. [37]) and have different statistics than place-tagged tweets. Other studies have looked at how geo-tagged tweets differ from non-geotagged tweets, e.g. Pavalanathan et. al. [38] found GPS-tagged tweets are written more often by young people and women, use more geographically specific words and are generally longer. There is also an extensive literature on the problem of geo-locating users based on inference from the user’s location field, words in their tweets or by locating based on their friends’ locations [39] [40] [41], [42]. However, all of our tweets have a ‘place’ tag, provided by the API, based on Twitter’s own location services. Since Twitter is able to access more information about each tweet than we can get from the API (e.g. GPS, cell tower signal or data about nearby wireless access points [43]) the location information provided in the place field is likely to be of good quality and we do not use any location inference methods.

### Creating a grid

We divide the geographical bounding box into a *X* × *X* grid. Since our original bounding box is not square, these grid cells are rectangular. As some grid boxes are over coastal areas, not all grid boxes cover the same land area. Let *A* be the land area of a grid box. To calculate *A* the shape-files for the UK were obtained from the GADM database of Global Administrative Areas [44]. *A* is calculated by projecting the UK polygon from WGS84 co-ordinates to an equal area projection and using standard planar techniques for calculating polygonal area.

Our choice of grid is arbitrary, we could just as easily have covered the collection area with hexagons or some other shape. We will show that our results are robust across a range of grid sizes, so that we actually have a scaling ‘law’ holding over a certain distance scale that does not depend on how the areas are chosen.

### Measuring tweet and user density

Let *N*_*t*_ be the number of tweets in a grid box, *N*_*u*_ the number of users in a grid box, *N*_*p*_ the population. We define

$$\begin{array}{cc} T & =N_{t}/A\\ U & =N_{u}/A\\ \rho & =N_{p}/A \end{array}$$ (1)

where *T* is the number of tweets/km^2^, *U* is the number of users/km^2^ and *ρ* is population/km^2^.

Each tweet is located, via the geo-tag or place tag, to either a bounding box given in the ‘place’ metadata or a single point given by a zero area bounding box or a GPS co-ordinate. When the tweet is located at a single point we add 1 to the count for the grid box containing that point. When the tweet is localised in a bounding box we add to every overlapping grid box, indexed by *b*, a fractional value:

$$f_{jb}=\frac{\text{Area}(\text{bounding}\;{\text{box}}_{j}\cap \text{grid}\;{\text{box}}_{b}\text{)}}{\text{Area}(\text{bounding}\;{\text{box}}_{j})}$$ (2)

to the count of tweets in that box, where *j* labels the tweet.

To count the users in a grid box we first count the number of tweets per user. For a user *i* let *N*_*t*_(*i*) be the number of tweets by that user. Since users can post from multiple grid boxes we split the contribution of each user proportionally across the places they tweet from. So, for each of user *i*’s tweets, labelled *j*, we calculate *f*_*jb*_ as above, for all overlapping grid boxes *b*. We add $\frac{f_{jb}}{N_{t}\left(i\right)}$ to the count of number of users in *b*. Thus if a user only ever tweets from within a single grid box we will end up adding 1 to the count of users in that grid box, if a user divides their time between two locations equally we will add 0.5 to the count of users in each grid box, and so on.

To measure the population in a grid box we used the latest UK mid-year population estimate [45] in each Lower Super Output Area (LSOA). LSOAs are polygonal areas designed by the Office for National Statistics to improve the reporting of small area statistics in the UK. They contain at least 1000 inhabitants with a mean of 1500 and are designed to be as consistent as possible in population size. They range in area from very small (smallest is ∼ 0.05 km^2^) to very large (largest is ∼ 250 km^2^) as we move from cities to the countryside. We downloaded the LSOA polygons from the UK Data Service [46]. When an LSOA intersects multiple grid boxes we divide the population in the LSOA among the intersecting boxes proportionally to the area of the intersection with the LSOA. When multiple LSOAs lie in the same grid-box we sum the intersecting LSOA populations. Around 80% of LSOAs are smaller than our smallest grid box (∼ 5 km^2^). Although the population within a LSOA is not necessarily uniformly distributed, the LSOAs are typically small enough that this is not a concern for our analysis, though it may result in some minor artefacts in rural areas when using very small grid boxes.

## Results

### Data collection

We collected 33,952,082 tweets in total during our collection period. We first removed 6 very active automated accounts (bots) whose tweets make up more than 1% of the total number of tweets in our dataset. These accounts are linked to e.g. automatic weather stations, solar panels, or phone number services that produce a huge number of geo-tagged tweets. The remaining tweets consisted of 2,051,250 tweets geo-tagged tweets with GPS co-ordinates inside the target area and 18,421,520 place-tagged tweets with bounding boxes contained in the target area. The remaining tweets either had geo-tags outside the target area or place-tag bounding boxes not fully contained inside.

There were 161,658 users who made at least 1 geo-tagged tweet and 370,545 users with at least one place-tagged tweet, with some overlap between these sets. When processing the data, if a tweet is geo-tagged we use that as the tweet location. If not we check the place_type field. If it is ‘country’ (e.g. United Kingdom) or ‘admin’ (e.g. South-West), we discard it since it does not provide sufficient precision. Otherwise we use the bounding box associated with that tweet for the location. The sampled users are a mix of individual users and organization accounts.

### Data fitting

We make the ansatze:

$$\begin{array}{c} T=CU^{\gamma }\\ U=B\rho^{\beta }\\ T=A\rho^{\alpha } \end{array}$$ (3)

Here *α*, *β*, *γ* are scaling exponents which will be crucial for us in what follows, while *A*, *B*, *C* are proportionality constants. A naive model might assume all people are equally likely to become Twitter users and all users are equally likely to author a tweet in a given time period. This would make the number of users directly proportional to the population (so *β* = 1) and number of tweets per user a constant (so *γ* = 1). Deviations from these values tell us about the actual adoption rates of Twitter and the behaviour of Twitter users. We find the actual values for *γ*, *β*, *α* by fitting to our data.

We note that in contrast to previous work, [20] [27] [28], here we use population density, rather than the total population associated with a town or city, as the independent variable. As we have discussed above, this allows us to use arbitrary sampling areas rather than being reliant on definitions of ‘city’ or ‘town’ and the geographical areas associated with each urban area. Population density has a number of other advantages. First, it lets us test the robustness of our results under changes in measurement scales (i.e. different grid resolutions). Second, it all ows inclusion of sparsely populated and/or un-named rural areas. Third, it thereby allows study of the entire South West UK region, rather than the sub-sample given by named towns and cities. Fourth, it includes space as an explicit factor within the modelling framework, opening up study of potential explanatory mechanisms that involve spatial processes.

If population density is the only relevant variable then by consistency we should have:

α=βγ. (4)

Deviations from this relation imply other variables are important for prediction of Twitter adoption and Twitter use in an area.

#### Place-tagged tweets

Fig 1 shows fits to Eq 3 for a 40 × 40 grid, using place tags to locate tweets and users. The fits were performed using linear least squares to fit the log-transformed data. Each point corresponds to a grid box with at least one tweet and one resident. The bottom right histogram shows that the majority of the log-transformed data lies in a narrow range, so the fit is most constrained there. We have attempted fits using lower and upper thresholds, as well as different binning schemes, which give similar results. We have used simple least squares fits of all the available data in order to avoid introducing any arbitrary scales into our results, since, as we will see later, a key part of our argument will be to discover the measurement scales where this approach is valid. All of the exponents are (statistically) significantly greater than one and the *R*^2^ values are relatively high, see the section **Deviations from scaling relationships** for more detail.

Fig 2 shows a range of different grid sizes (finer grids on the right) demonstrating that the exponents are consistent for a range of grid sizes, approximately *X* = 32 to *X* = 80, corresponding to physical sizes roughly 82km^2^ to 13km^2^. We will call this the ‘scaling window’. The plot of tweet density, *T*, versus population density, *ρ*, in Fig 1 shows the biggest deviations from a power law relationship at low population density, indicating that at low population density other factors determine the number of tweets and users. We will discuss this deviation in the following sections.

#### Geo-tagged tweets

**Fig 3:**
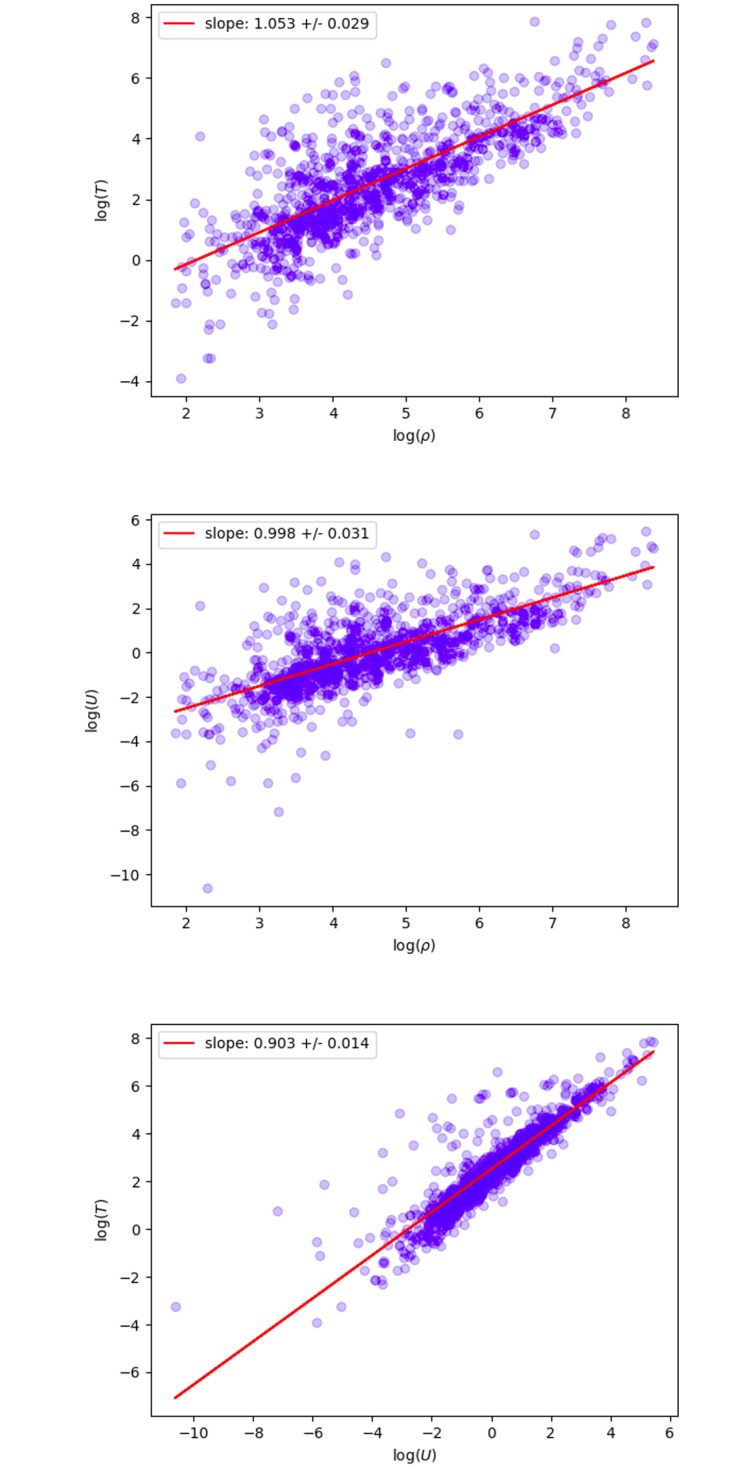
Scaling laws for geo-tagged tweets. Left: Tweet density *T* versus population density *ρ*, slope of the line is *α*. Middle: User density *U* versus population density *ρ*, slope of the line is *β*. Right: Tweet density *T* versus user density *U*, slope of the line is *γ*. Using a 40 × 40 grid.

**Fig 4:**
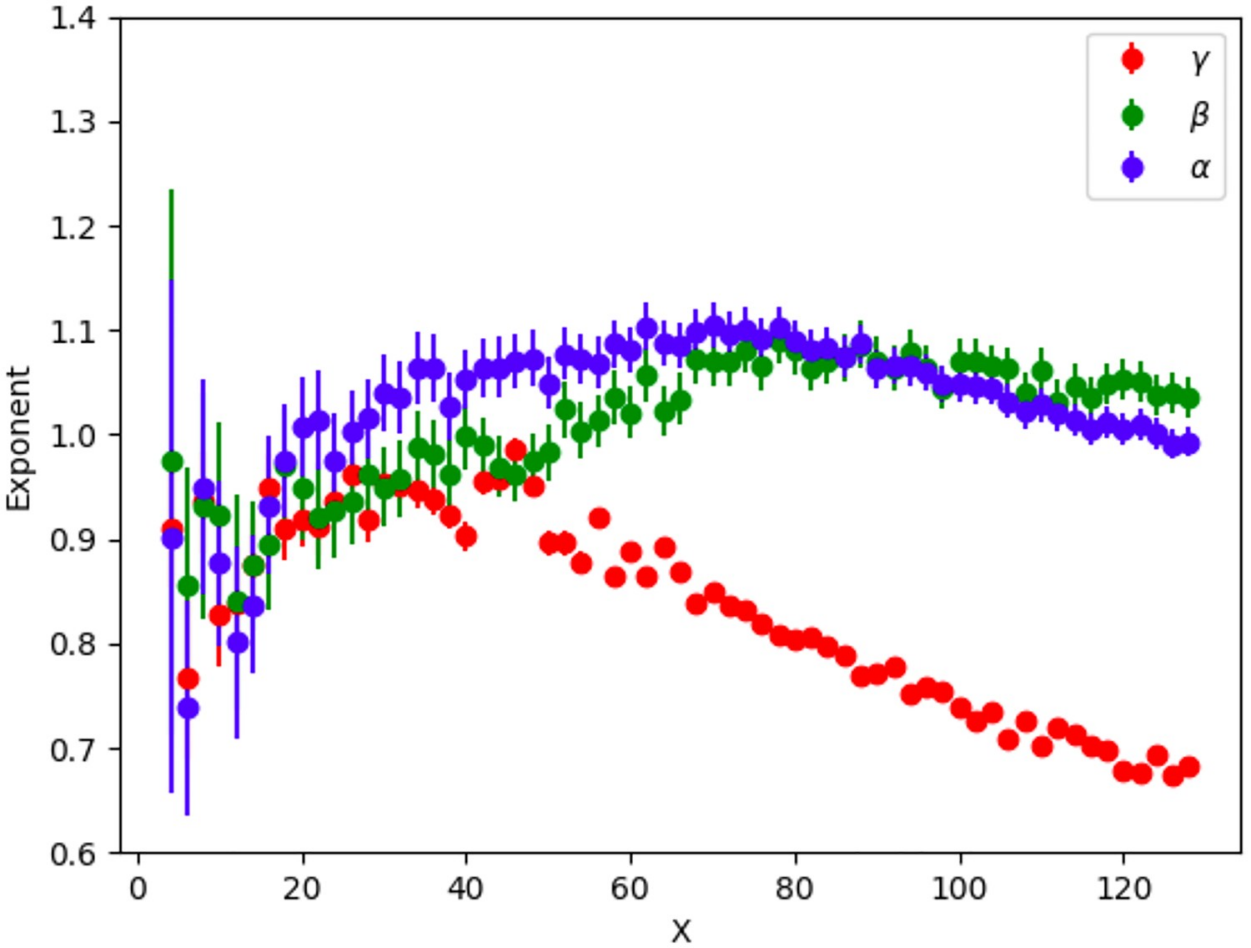
Exponents *α*, *β*, *γ* versus grid size *X* for geo-tagged tweets.

Fig 3 shows fits to Eq 3 for a 40 × 40 grid, using geo-tags to locate tweets and users. Each point corresponds to a grid box with at least one GPS tagged tweet and one resident. The exponents *α* and *β* are consistent with one, while the exponent *γ* is less than one. Fig 4 shows a range of different grid sizes. The exponents, especially *γ*, vary a lot as the spatial scale changes. There is a small range of grid sizes, X = 40 to X = 64, where the exponents are approximately constant (corresponding to physical sizes roughly 52km^2^ to 20km^2^). However this relationship does not seem to be as robust as that found for place-tagged tweets. Most importantly, though we find reasonably accurate fits to the data, the exponents are all significantly smaller, with the tweets per user exponent, *γ*, less than 1. These facts indicate that geo-tagged tweets are markedly different than place-tagged ones. We will discuss possible reasons for this in the following section.

### Source of tweets

Twitter provides a field in the meta-data associated with each tweet: ‘source’. This records the utility or application that was used to post the tweet e.g. ‘Twitter for Android’. For tweets with geo-tags and tweets with only place-tags we rank the most common sources in Tables 1 and 2. These two tables can explain much of the difference between geo-tagged and place-tagged tweets.

**Table 1:** Top 5 sources of geo-tagged tweets in our data set. There are 2,051,250 tweets in total.

Rank	Source	Proportion %
1	Instagram	61.2
2	Sandaysoft Cumulus	5.8
3	Foursquare	4.0
4	dlvrit.com	3.6
5	dlvr.it	3.4

**Table 2:** Top 5 sources of place-tagged tweets in our data set. There are 18,421,520 tweets in total.

Rank	Source	Proportion %
1	Twitter for iPhone	60.9
2	Twitter for Android	22.9
3	Twitter Web Client	11.3
4	Twitter for iPad	3.5
5	Tweetbot for iOS	1.1

Looking at Tables 1 and 2, we see very different sources for the different types of tweet. The majority of tweets with geo-tags are Instagram posts that have been shared on Twitter. Sandaysoft Cumulus [47] is software for personal weather stations, dlvrit [48] is an automated social media service for marketers and Foursquare [49] is a social media platform for consumer recommendations. Thus geo-tagged posts are predominantly shared posts from other websites (Instagram and Foursquare), automated weather bots and marketing accounts. In contrast, place-tagged posts typically originate from Twitter clients on mobile devices and the Twitter website itself.

Instagram is itself a very active and popular social media platform, making it likely that users would respond to Instagram posts directly on Instagram, rather than via Twitter. It is also unlikely that bots (of the kinds found here) will reply to other bots. To check this we can examine the tweet meta-data fields ‘in_reply_to_status_id’, ‘in_reply_to_user_id’ and ‘quoted_status_id’. If either of the first two fields are non-empty the tweet is a reply to another user, if ‘quoted_status_id’ is non-empty the tweet quotes another tweet. Twitter also has ‘retweets’; however, requesting tweets by location from the API, as we did, returns no retweets, presumably as the retweeter’s location may be different than the location of the original tweet. Thus we can only see replies and quotes in our dataset. Of the 2,051,250 geo-tagged tweets, 98,029 are replies or quotes, making up 4.8% of the total. In contrast, for the 18,421,520 place-tagged tweets there are 9,299,621 replies or quotes, making up 50.4% of the total.

Clearly tweets with geo-tags are qualitatively different from tweets with place-tags, in both the kind of user they originate from and the content they contain. Since geo-tagged tweets are mostly either from bots or from users sharing social media content from other platforms, these tweets do not seem to represent the kind of Twitter-native human social interaction that researchers appear to be looking for when they study Twitter. From here on we will only consider place-tagged tweets, since these seem to better represent the kind of Twitter-native data that most researchers intend to study.

### Checking the consistency relation

**Fig 5:**
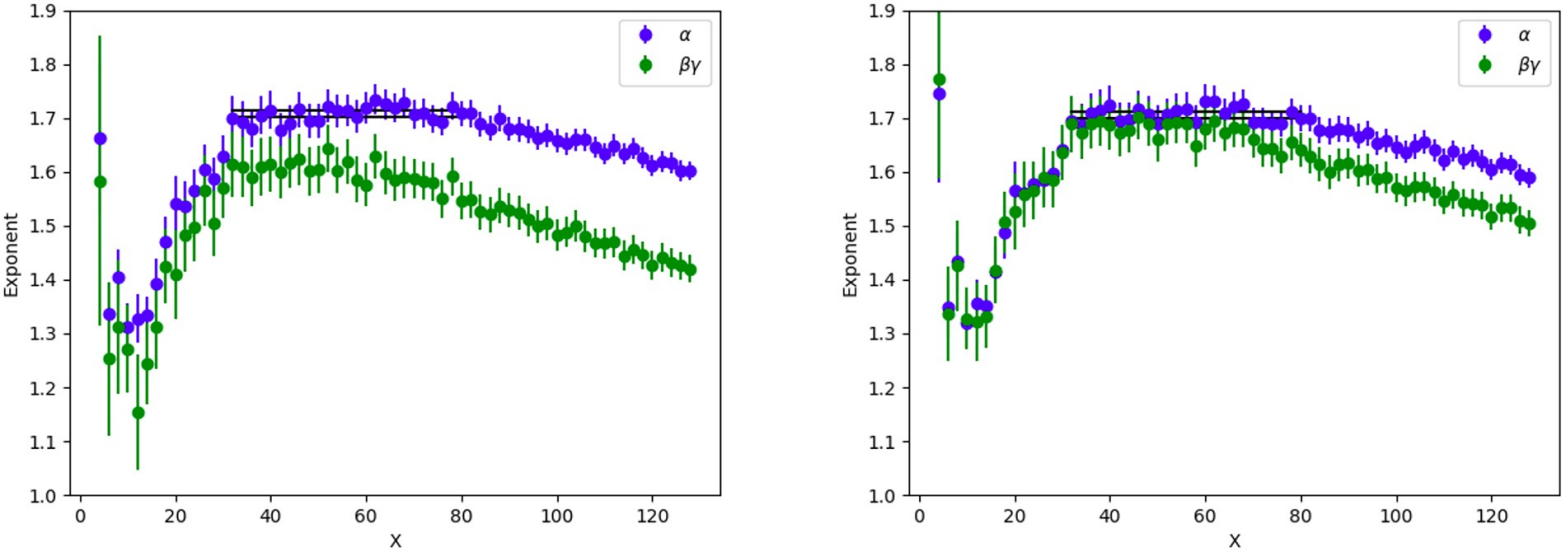
Consistency relation check. Left: Comparing the exponent *α* obtained from fitting the data to *βγ* using all the place-tagged tweets. Right: Comparing the exponent *α* obtained from fitting the data to *βγ* using only users with at least 10 place-tagged tweets during the data collection period. Black line is the average of *α* across grid sizes from *X* = 32 to *X* = 80.

We check the consistency relation give in Eq 4 in Fig 5. We find some disagreement, indicating that residential population density is not the only factor accounting for the number of tweets per user. We get better agreement by looking only at users who have tweeted multiple times in our observation region over the course of the study. For example, Eq 4 is more closely satisfied if we restrict our fits to users who made at least 10 tweets during the whole observation period. This reduces our count of 14,210,527 tweets from 311,843 users to 13,649,169 tweets from 114,565 users. Since it is more likely that users who tweet often in a region live there, and so are counted in the census, the improved agreement is to be expected. The lack of exact agreement is not surprising; multiple studies [14] [16] have shown that gender, race, age and other demographic variables are predictive of Twitter use, which are not captured here. Indeed it is surprising that population density alone does such a good job of predicting user density and tweet density. We explore the difference made by population mobility (e.g. commuting) in S2 Text.

### Deviations from scaling relationships

**Fig 6:**
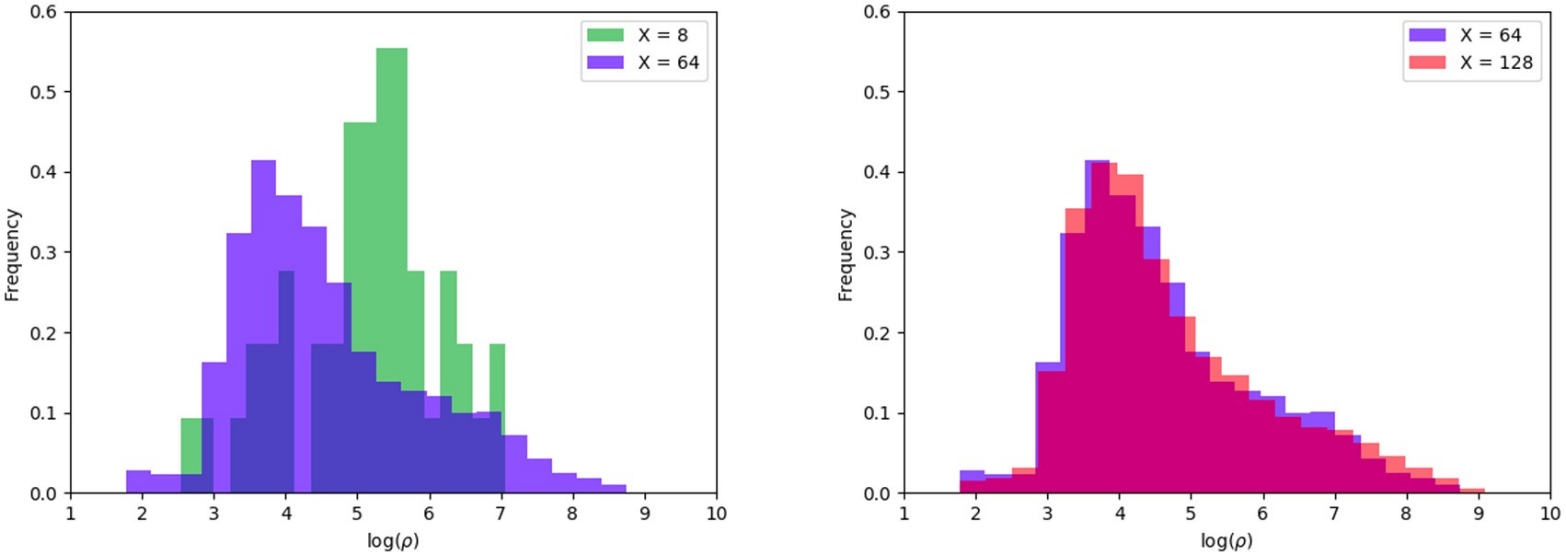
Histogram of log(*ρ*). Comparing an 8 × 8 grid and a 64 × 64 grid on the left and a 64 × 64 with a 128 × 128 grid on the right. The 128 × 128 and 64 × 64 grids give similar distributions with long tails while the 8 × 8 grid is concentrated around a central value.

For large grid boxes (e.g. *X* < 32), the exponents fall off rapidly, Fig 2. This is because using very large grid boxes does not generate a diverse sample of homogeneous grid boxes that each represent a different area class (e.g. rural, suburban, urban). Instead large grid boxes are likely to capture a heterogeneous mixture of rural and urban areas and average them together. Smaller boxes are more likely to capture a homogeneous area (e.g. rural or urban, but not both). This effect can be seen by plotting a histogram of population density for large, intermediate and small grid boxes, Fig 6. We see that the distribution of *ρ* over large areas (e.g. 8 × 8) is qualitatively different to the distribution over smaller areas (e.g. 64 × 64, 128 × 128).

**Fig 7:**
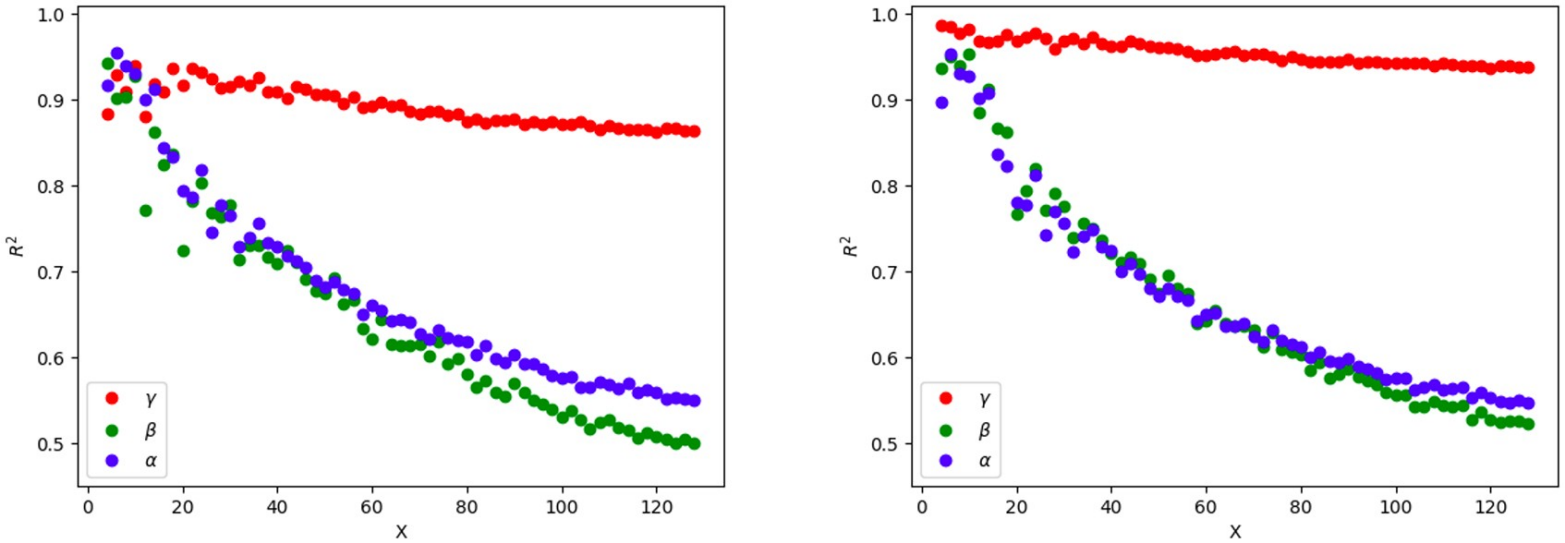
Fit quality check. Coefficient of determination *R*^2^ for each fit of the data to Eq 3 across a range of grid sizes. Left: Fits using all place-tagged tweets. Right: Restricting the fits to users with at least 10 tweets. Tweet density *T* is very well predicted by user density *U*. Across the scaling window from *X* = 32 to *X* = 80, between 75% and 60% of the variance in *T* and *U* is predicted by population density *ρ*.

Exponents also fall away for small grid boxes, *X* > 80. This is because very small boxes may contain too few people to be treated as ‘populations’. We see this reflected in a decreasing fit quality as the grid resolution increases. Fig 7 shows the coefficient of determination, *R*^2^, for each fit on each grid. *R*^2^ is the proportion of the variance in the dependent variable that is predictable from the independent variable. For predicting *T* given *U* we get *R*^2^ greater than 0.9 for most grid sizes. The *R*^2^ value falls off more rapidly with decreasing grid box sizes for fits of *T* and *U* against *ρ*. As grid boxes get smaller, local and historical explanations are necessary to predict Twitter usage. In the limit, when the boxes become small enough to contain only a handful of users, clearly a model based on the characteristics of individuals rather than areas is more appropriate. This is reflected in the decreasing *R*^2^ for the *α* and *β* fits and the deviation from scaling for finer grids, *X* > 80.

### Scaling summary

Given the results above, Twitter activity belongs to the large class of social outputs (such as patents [21], homicides [31] and bank transactions [27]) that scale super-linearly with population. In contrast to the references cited, and to most work on scaling laws, we do not use the absolute population sizes of individual cities as units of analysis. Instead we choose gridded spatial sampling areas and use population *density* as the size variable. This allows us to examine the physical scales where the scaling relationship holds and where it breaks down. Our results suggest that the statistics of very large areas (e.g. counties) or very small areas (e.g. neighbourhoods) are qualitatively different. Therefore behavioural models like [22] and [27] are most appropriate at the intermediate scales we have identified in this work.

## Finding anomalies

### Measuring anomalies

Given the robust trends observed in the place-tagged data we can predict the typical Twitter activity for an area fairly accurately, given its population density or the density of Twitter users. We can then ask about places which deviate most strongly from the observed trend. It is this deviation from the trend, rather than unusually high or low numbers of tweets, that one should use to identify an anomalous area. An area might have a very high or very low number of tweets, but if this number is well-predicted by Eq 3, then this area is ‘typical’. If it has many more or less than expected we might inquire further into the possible causes.

We will call the difference between predicted tweet density *T* = *CU*^*γ*^ and measured tweet density $\tilde{T}$ the ‘anomaly’:

$$A_{TU}=\tilde{T}-CU^{\gamma }$$ (5)

Large positive values of *A*_*TU*_ indicate many more tweets than expected, large negative values indicate many fewer. We could define the other anomalies *A*_*Tρ*_ and *A*_*Uρ*_ similarly. Examining Figs 1 and 7 we see that fits of *T* against *U* are most precise. Until we have a more precise model for *U* and *T* as a function of *ρ* the anomalies *A*_*Tρ*_ and *A*_*Uρ*_ would be likely to simply measure fitting error. For *T* versus *U* the simple power law relationship fits the data very well across all scales, so we claim that *A*_*TU*_ is measuring something significant about the area.

We can also normalise the anomaly:

$${\hat{A}}_{TU}=\frac{\tilde{T}-CU^{\gamma }}{\sqrt{CU^{\gamma }\tilde{T}}}$$ (6)

and measure relative deviation from the fit instead of absolute deviation. The absolute anomaly will tend to emphasise cities and towns, since they generate more tweets and have proportionally larger deviations, whereas the relative anomaly ${\hat{A}}_{TU}$ controls for population density and puts all areas on an equal footing. Relative deviation will classify a rural area with 4 tweets observed where 2 tweets were expected as equivalently anomalous to a town with 40,000 tweets observed where 20,000 tweets were expected.

Other work on city scaling pursues similar tactics. Alves et. al [31] use distance to the scaling law, equivalent to Eq 5, while Bettencourt et. al. [21] define a metric that they call a SAMI (Scale Adjusted Metropolitan Indicator) which is again equivalent to Eq 5. The aim of a scale adjusted metric is simply to provide a fair comparison between different areas. With Twitter now used to study phenomena from disaster relief [50], to public health monitoring [51], to crime prevention [52], knowing how population density affects baseline Twitter activity is crucial. Linear correlation will fail to predict the correct relationships between Twitter activity and other variables and this will have implications for public policy, if/when insights drawn from social media start to inform policy makers.

### Mapping anomalies

**Fig 8:**
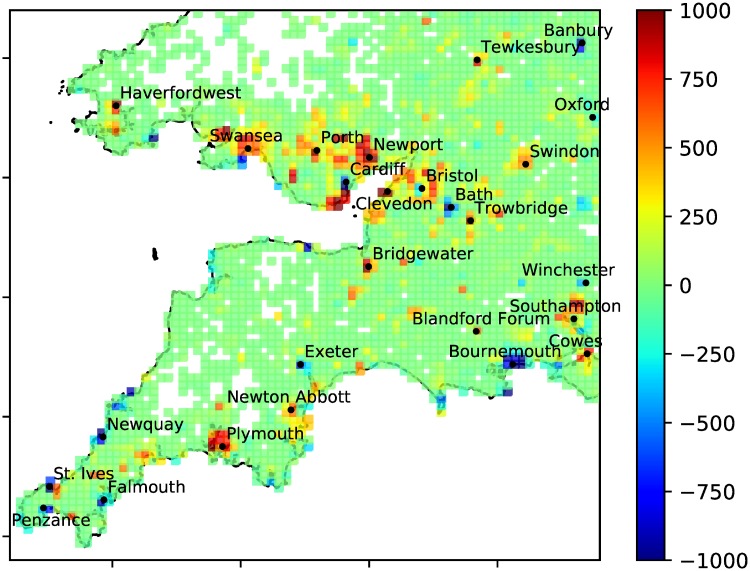
Absolute anomaly. *A*_*TU*_ plotted as a heatmap over the South-West UK (with |*A*_*TU*_| capped at 1000 for better resolution). White space indicates less than one tweet or less than one person per km^2^ in the grid box. 80 × 80 grid.

**Fig 9:**
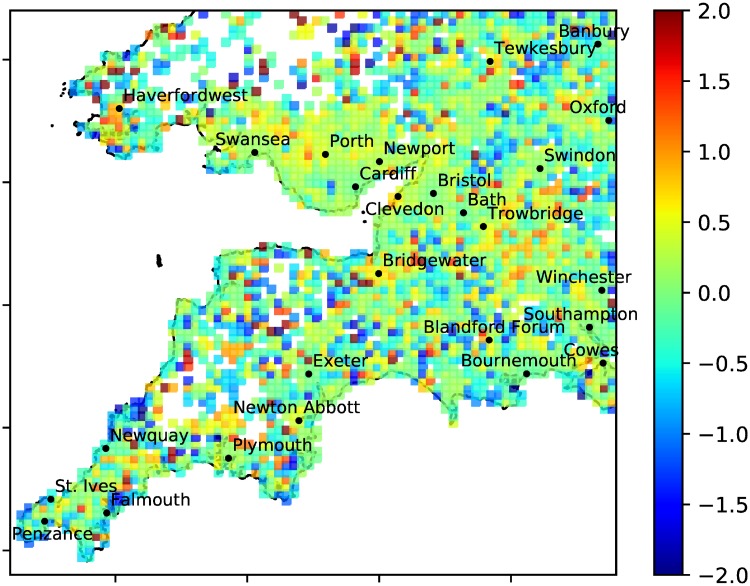
Relative anomaly. ${\hat{A}}_{TU}$ plotted as a heatmap over the South-West UK (with $|{\hat{A}}_{TU}|$ capped at 2 for better resolution). White space indicates less than one tweet or less than one person per km^2^ in the grid box. 80 × 80 grid.

Using the place-tagged tweets from users who tweeted at least 10 times during the collection period, Fig 8 shows *A*_*TU*_ and Fig 9 shows ${\hat{A}}_{TU}$. The absolute anomaly *A*_*TU*_ emphasises towns and cities as expected. In Fig 8 we see some towns with deficits (negative anomalies) like Exeter, Bournemouth and Cardiff and some with excesses (positive anomalies) like Newport, Plymouth and Southampton. Looking at the relative anomaly ${\hat{A}}_{TU}$ there is no obvious pattern of positive or negative relative anomalies associated with the countryside or towns and cities, i.e. we have successfully detrended the tweet density data. Local hotspots might be examined to discover their causes (e.g. tourist attractions or festivals), but we do not attempt this here.

### (Not) Explaining anomalies with youth

**Fig 10:**
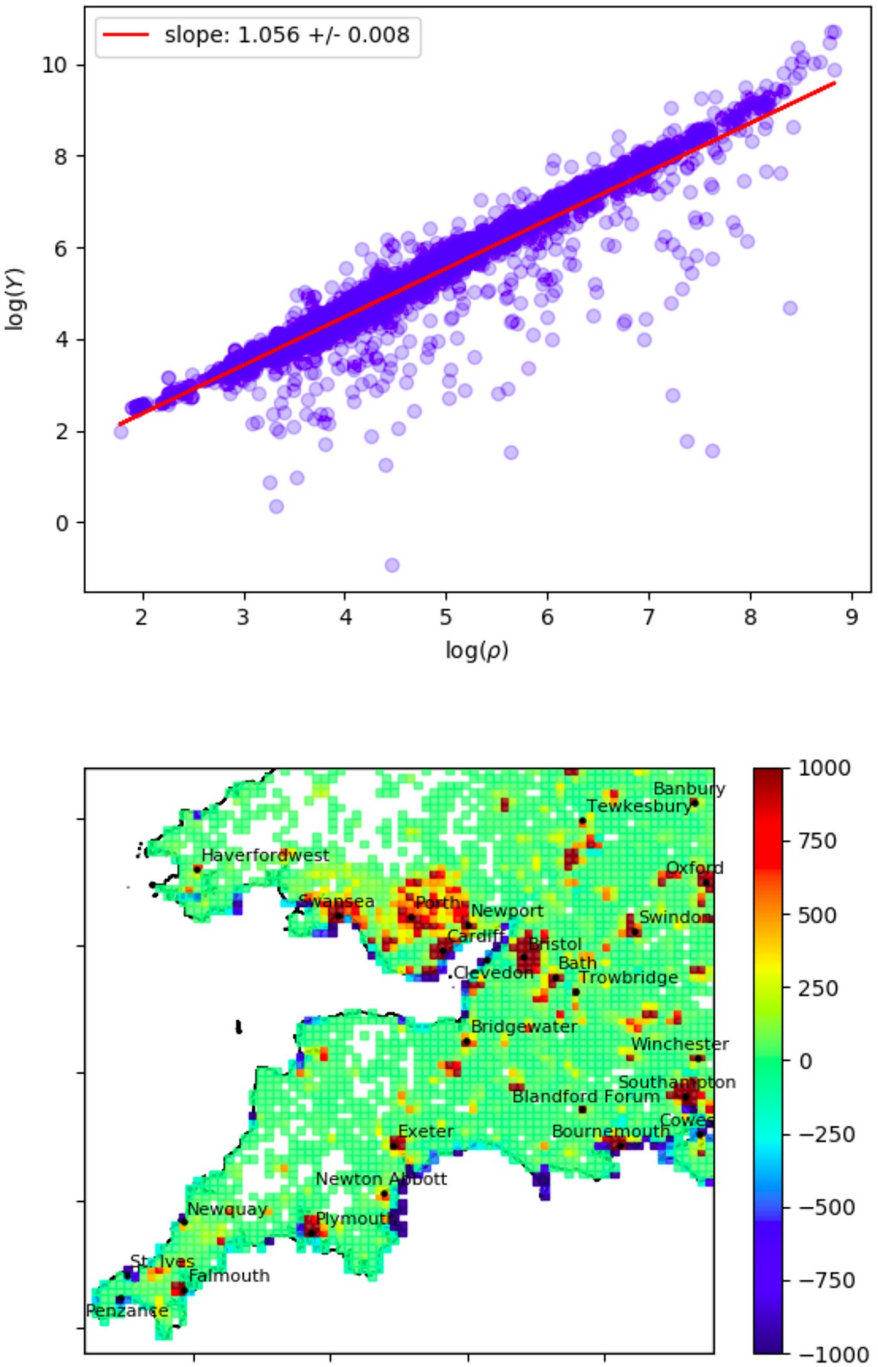
Population anomaly. Top: Population density *ρ* versus youth-population density *Y* on a log-log axis. Bottom: *A*_*ρY*_ plotted as a heatmap over the South-West UK (with |*A*_*ρY*_| capped at 1000 for better resolution). This anomaly measures the excess or deficit of the youth population after accounting for the non-linear scaling above. Here we show the same grid boxes (80 × 80) as Fig 8.

As previous work has shown a bias towards young people in Twitter usage, and since this information is also recorded in the census, here we attempt to relate the relative youth of a local population to deviation from the tweet density trend. Plotting the number of people aged 18 to 35 per unit area *Y* against the population density *ρ* on a log-log scale we find another power law relationship. Fig 10 shows the census data fit to the ansatz

$$Y=D\rho^{\delta }$$ (7)

This indicates that there are proportionally more young people in densely populated areas of the South-West UK. We can define the absolute and relative anomalies as before using the measured ‘youth’ $\tilde{Y}$ and the predicted value *Y* = *Dρ*^*δ*^,

$$A_{Y\rho }=\tilde{Y}-D\rho^{\delta }$$ (8)

$${\hat{A}}_{Y\rho }=\frac{\tilde{Y}-D\rho^{\delta }}{\sqrt{D\rho^{\delta }\tilde{Y}}}$$ (9)

**Fig 11:**
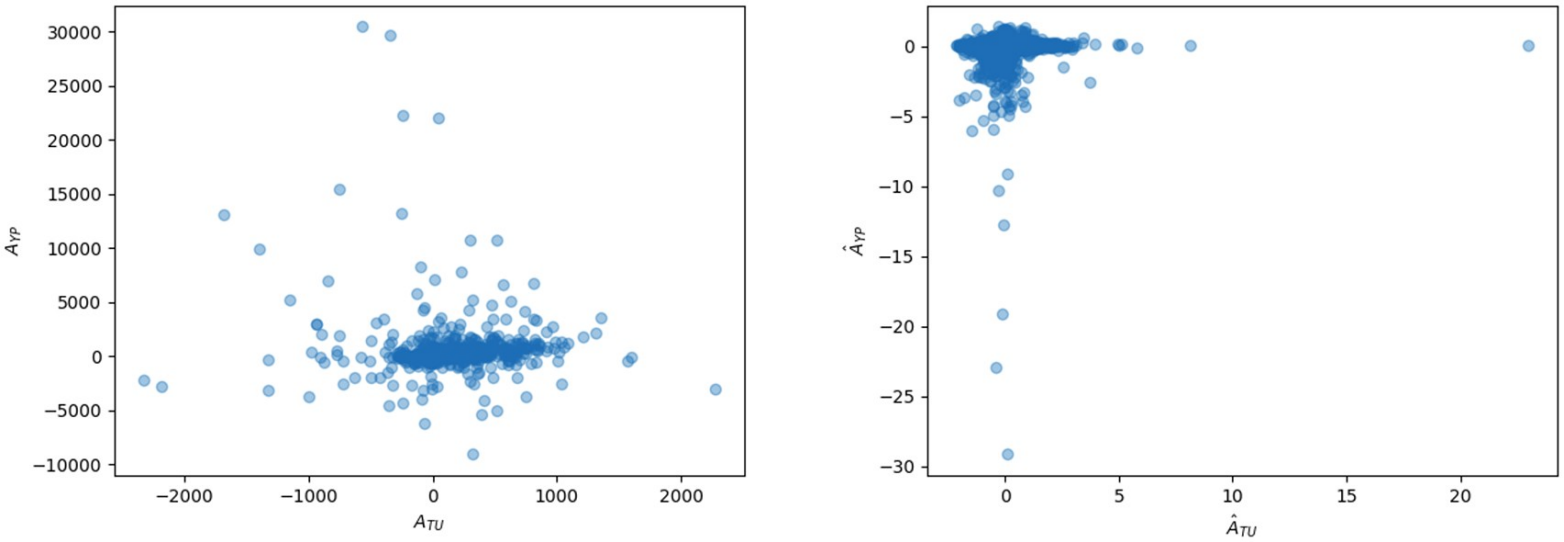
Comparing anomalies. Left: Plotting absolute anomalies in young people, *A*_*Yρ*_, against absolute anomaly in tweets, *A*_*TU*_. Right: Plotting relative anomalies in young people, ${\hat{A}}_{Y\rho }$, against absolute anomaly in tweets, ${\hat{A}}_{TU}$.

We plot the absolute and relative anomalies for both youth and tweet density against each other in Fig 11. Neither of the observed relationships is particularly strong. The absolute anomalies have a roughly linear relationship, with many outliers; surprisingly the plot suggests a negative relationship, which would indicate that an excess of young people predicts a *deficit* of tweets. The relative anomalies show no clear relationship. What these plots demonstrate well is that, after accounting for the common correlate of population density, the relationship between anomalies is much weaker than the relationship between the raw quantities. We conclude that in our data set, youth is only indicative of high Twitter activity due to the common association of both variables with population density. Demonstrating that youth (or other demographic variables, like income or ethnicity) has any additional effect beyond this is a more difficult question and we do not pursue it here. As the relative youth of a population has often been posited as an explanatory variable in accounting for Twitter activity on the basis of linear-correlation [16], this is already a significant result. The broader implication is that the relationship between Twitter activity and demographic variables cannot be determined by simple linear correlations.

## Discussion

We have investigated relationships between population, user and tweet density. Using place-tagged tweets, we find a robust power law relationship between these quantities that holds across a range of measurement scales with the same exponent. When we look at users who tweet often in our sampling area, and so probably live there, population density does a good job of predicting how many users and tweets we should see in an area. Unusual areas can be identified by looking at deviations from the power law relationship and can have high or low numbers of tweets and users.

Our fits of user density against population density using place-tagged tweets yield exponents, *α*, *β* and *γ* greater than one. There is a range of distance scales over which the exponents are constant giving us confidence that these exponents are not artefacts of the sampling procedure. Super-linear scaling laws like these are often observed for social phenomena [19] [20] [26], where increasing the density of people generates synergistic effects that encourage richer and more active social, scientific and business networks. We observe the same thing here on Twitter.

We do not see these effects when using geo-tagged tweets and have shown that geo-tagged tweets are very different in nature to place-tagged tweets. Geo-tagged tweets often originate from automated accounts or via shares from other social media platforms. Due to the large number of studies utilizing geo-tagged tweets this is an important consideration for future research.

Our study builds on previous studies which ask about sampling biases in Twitter data. Those studies consistently find a bias towards urban areas, i.e. areas with large population density. We have demonstrated that this systematic bias can be modelled with a simple power law function. The number of tweets grows super-linearly with number of users, in line with work on other scaling laws in cities. Since Twitter is such a rich and useful data source we believe this study will enable better, more representative, sampling of tweets and a better interpretation of research based on Twitter.

We also believe that social media data can be a powerful test of models developed to explain super-linear scaling e.g. [21] since the underlying social network can often be discovered and the large volumes of data should give high statistical precision. Twitter data is particularly suited for this purpose, being free and open, unlike other potential data-sources like phone call records, or Facebook pages. For example, our work is partially motivated by the work of [26] and [19] on social networks; as shown in the literature (e.g. [39]), online friendship networks are closely correlated with offline networks, which are spatially embedded. Furthermore, we might speculate that the spatial proximity of other people (i.e. population density) has an effect on ease and frequency of communication, and hence potentially on use of social media. Working with population densities, the observation of a scaling law like *T* = *Aρ*^*α*^ might be interpreted in terms of the effect of population density on interaction frequency, which is harder to infer from a similar relation with absolute population size. Contrast 10000 people spread across a large rural area with 10000 people in a small town. The population size is identical, but the likelihood of interaction is much greater in the town, which by the argument above might lead to more social media activity.

Finally, in section we have shown that while the ‘youth’ of an area (number of 18 to 35 year-olds) is correlated with high Twitter usage, when we correct for the common association of both variables with population density and correlate the residuals, the association vanishes. This is an important lesson for other demographic studies and here the non-linear relationship between tweets (*T*) and population (*ρ*) is key. If a linear relationship was assumed we would under-correct for the dependence of *T* on *ρ* and discover a spurious relationship between the variables.

In future we hope to extend this work in a number of directions by sampling larger areas including London and perhaps other ‘mega-cities’. It remains an open question as to whether these mega-cities obey the same statistics as the smaller cities examined in this work. Discovering similar relationships in other geographic areas e.g. the US, would add a lot of weight to these findings and strengthen the case for calling this a scaling ‘law’. Finally other geo-tagged social networks like Foursquare, Instagram or Sina-Weibo may display similar or different characteristics in the relationship between activity and population density. This work demonstrates several key methodologies and observational facts and provides a rigorous and useful basis further work as well exposing these scaling relationships and how they can be used to study trends in social media. As social media data comes to inform public policy in health, disaster relief, crime and a host of other key areas, a partial understanding of the data risks causing material harm to people’s lives and livelihoods. Properly accounting for bias in social media data is becoming not only a matter of scientific accuracy but also a social and political necessity.

## Supporting information

S1 Text Cross-validation. (PDF)

S2 Text Comparing working and resident population. (PDF)
