# Supplementary material for: Scaling laws in geo-located Twitter data
Source: PLoS One. 2019 Jul 24;14(7):e0218454. doi: 10.1371/journal.pone.0218454 (PMC6655604; doi:10.1371/journal.pone.0218454)
Supplement: S1 Text — (PDF) [file pone.0218454.s001.pdf]

S1 Text: Cross-validation.

To check that the arbitrary bounding box does not cause artefacts we randomly placed bounding boxes covering 25% of the area of our original bounding box within the original sample area. We then repeated the whole analysis using only tweets in the sub-boxes, repeating this process 1000 times to construct a resampling distribution. Grid resolution was maintained, for example, when sub-sampling with $X = 80$ the sub-area was covered by a 40×40 grid.

We compare the sub-sampled data to the full data set in Figure S1.1. Across the scaling window, $X = 32$ to $X = 80$, the sub-sampled exponents are consistent with the exponents calculated from the full data set.

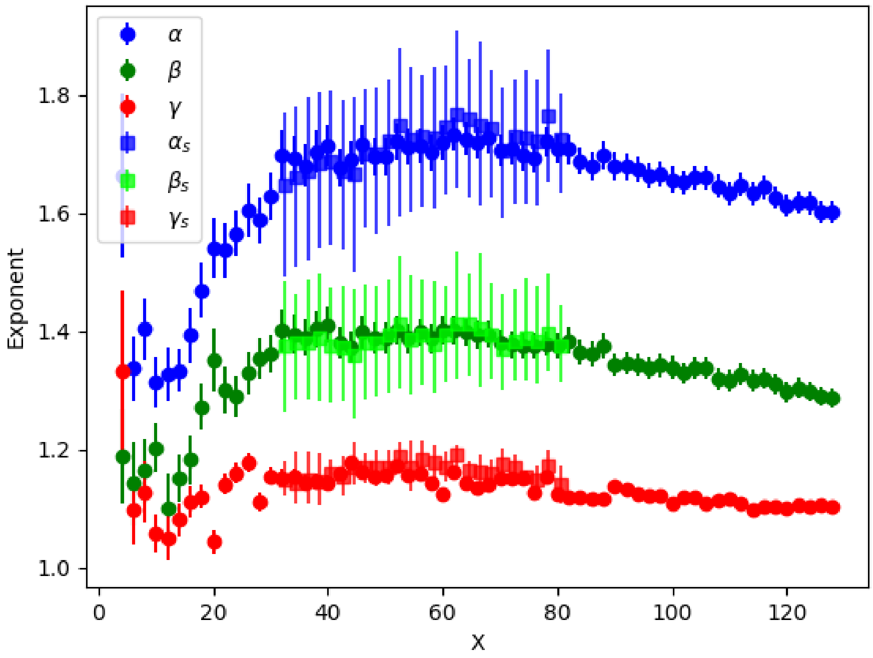

Fig S1.1 Bounding box cross-validation. Fits of exponents across a range of scales. Circles show the same data as Figure 1, i.e. exponents fitted using all available place-tagged tweets with error bars indicating the fit uncertainty. Squares show exponents fitted from 1000 sub-samples of an area 25% the size of the original bounding box, with error bars now showing the 68% confidence interval of the resampling distribution.

To check that the spatial proximity of our grid-boxes is not biasing our results, we repeated the analysis using a random subset of 5% of the populated grid-boxes. We performed this analysis 1000 times to create a resampling distribution for the fitted

exponents. We compare the resampling distribution to the original results in Figure S1.2. Figure S1.2 also shows the case where the random sampling is restricted to never choose two boxes sharing an edge or corner. In both cases we find the exponents are consistent with the full data set.

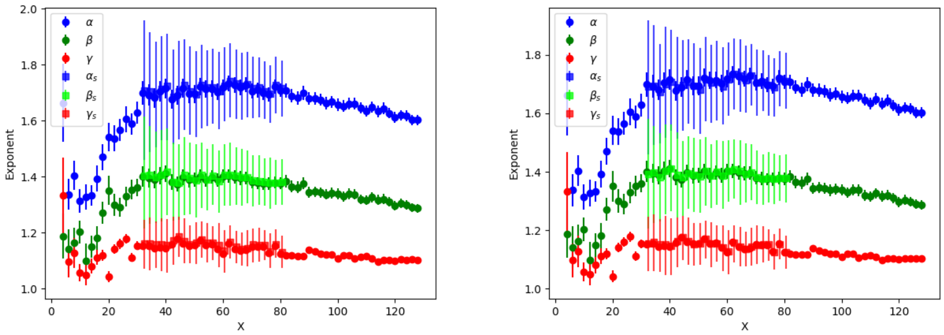

Fig S1.2 Grid sub-sampling cross validation. Fits of exponents across a range of scales. Circles show the same data as Figure 1, i.e. using all available tweets with error bars indicating the fit uncertainty. Squares show the same fits, using 5% of the data, where the error bars show the 68% confidence interval of the resampling distribution. Left: Allowing neighbouring boxes to be chosen. Right: Preventing neighbouring boxes from being chosen.
