# Supplementary material for: Scaling laws in geo-located Twitter data
Source: PLoS One. 2019 Jul 24;14(7):e0218454. doi: 10.1371/journal.pone.0218454 (PMC6655604; doi:10.1371/journal.pone.0218454)
Supplement: S2 Text — (PDF) [file pone.0218454.s002.pdf]

Scaling Laws in Geo-Located Twitter Data.

S2 Text: Comparing Working and Resident Population.

At the scale of census sample areas (LSOAs) workday and resident population levels can be dramatically different. The consistency relation check in Figure 5 measures this effect to some degree. Users are located based on where they tweet from, and can be spread across multiple grid boxes. Since we have determined that the relationship between tweets T and the resident population P is consistent with the relationship between T and the number of users U in our grid boxes, we have established that the daily movement of people does not strongly affect our conclusions (namely that a super-linear scaling law exists).

In order to directly examine difference, we have obtained workday and residential populations for the South-West UK from the 2011 Census [1]. Workday populations have not been extrapolated beyond the original census date, so we can only compare the populations as they were in 2011. As in our analysis above, we aggregate LSOAs into grid boxes and show workday population plotted against residential population in Figure S2.1 for our 32×32 grid ($\sim 82\text{km}^2$) and our 80×80 grid boxes $\sim 13\text{km}^2$. As the 80×80 grid is the lower bound of our scaling window and the finest grid we consider this is the most crucial plot.

The key observations are:

- There is a linear relationship between working and residential population when aggregated on the scale of our finest grid.
- The slope of the line is close to 1, 1.06 for the finest grid.

A slope of 1 would indicate that, at the aggregation level considered, working and resident populations were the same. The 6% or so deviation is statistically significant, and indicates that grid boxes with higher resident populations also contain more working locations and *vice versa* for boxes with lower resident populations.

Figure S2.1 also shows the line $P_{work} = P_{res}$. The bulk of the data is seen to be symmetrically distributed around this line, the positive slope of the best fit is likely due to high density outliers (e.g. city centres). This symmetric dispersion is further evidence that replacing residential with working population would make no substantial difference to our conclusions.

These considerations, together with the consistency relation check in Figure 5, give us confidence that the daily movement of people does not alter our qualitative conclusions (observation of a scaling law) and does not significantly alter our quantitative results.

[1] https://www.nomisweb.co.uk/census/2011

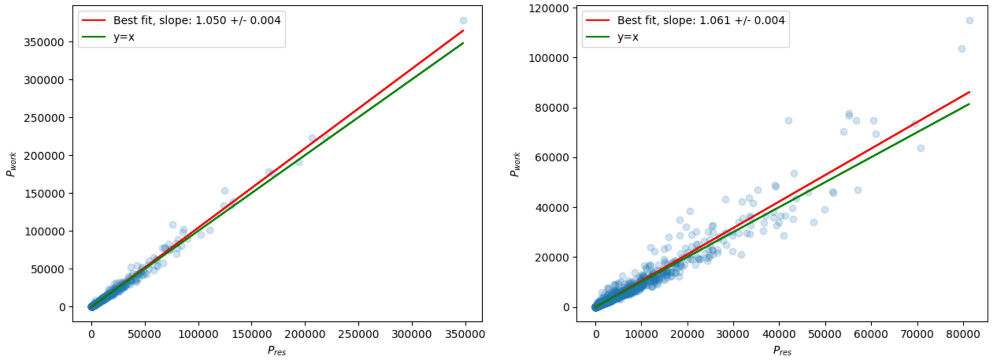

Fig S2.1 Working versus residential population comparison. Left plot shows 32×32 grid, right shows 80×80 grid. R^2 is 0.989 for left plot and 0.944 for right plot.
